# Editorial: The impact of psychological well-being on university students

**DOI:** 10.3389/fpsyg.2025.1684619

**Published:** 2025-11-25

**Authors:** Cristina Torrelles-Nadal, Yun-Chen Chang

**Affiliations:** 1 National Institute of Physical Education of Catalonia, University of Lleida, Lleida, Spain; 2 China Medical University, Taichung, Taiwan

**Keywords:** psychological wellbeing, university students, mental health promotion, protective factors, positive psychology interventions

University life represents a critical period of transition and transformation. While it offers opportunities for intellectual and personal growth, it also presents a wide range of psychological stressors that can affect academic performance, social functioning, and long-term mental health. In recent years, growing concerns over university students' wellbeing have prompted researchers and institutions to explore proactive strategies for supporting mental health in academic settings, recognizing that psychological wellbeing influences not only academic performance but also social adjustment and long-term mental health.

This Research Topic brings together empirical and theoretical contributions that deepen our understanding of how psychological well-being can be supported, maintained, and enhanced in university students through diverse perspectives and methodologies. These contributions identify risk factors and protective mechanisms and provide evidence-based interventions that promote mental health and resilience among university populations. These articles cover a wide range of themes and are synthesized here into key areas, beginning with those addressing the pervasive impact of stress, academic pressure, and emotional distress.

## Stress, academic pressure, and emotional distress

A recurring theme across the contributions is the pervasive impact of stress on university students. Academic pressures, financial concerns, and social isolation are consistently associated with elevated levels of anxiety and depression. Beroíza-Valenzuela offered a Chilean perspective, showing how sociopolitical events and the pandemic have intensified academic stress and widened mental health disparities. Kotnik et al. demonstrated that academic stress and mental fatigue impair student engagement. Wang et al. applied the Health Belief Model to identify psychological barriers to help-seeking among Chinese undergraduates. Alsalman et al. reported that stigma and attitudinal barriers limit the use of mental health services among Saudi medical students. Torrelles-Nadal et al. demonstrated how emotional intelligence and gratitude can buffer stress and foster adaptive coping strategies. Chen et al. found that positive emotions protect deaf students from the negative effects of perceived stress on academic engagement. Picker-Roesch et al. revealed that early academic stress predicts reduced career ambitions, especially among women. Heumann et al. documented the high rates of depression and anxiety among German students during the latter stages of the COVID-19 pandemic, with financial strain and low social support as key contributing factors. Parrello et al. linked reduced confidence in the future to higher distress, while optimism and a sense of meaning can mitigate these effects. Cui et al. highlighted resilience as both a mediator and a moderator between personality, self-esteem, and wellbeing. Bekesiene linked conscientiousness, openness, and extraversion to higher academic and military performance through self-efficacy. Wu et al. demonstrated that self-acceptance bridges the gap between social support and meaning in life, acting as a protective factor. Moussa-Chamari et al. associated poor sleep, depression, and low physical activity with a reduced quality of life. Guo proposed arts education as a pathway to enhance self-efficacy, resilience, and emotional intelligence. Güler Öztekin identified internet addiction as a risk factor for school engagement, mediated by depression, anxiety, and stress.

Beyond individual coping mechanisms, these studies also highlight the role of broader protective factors that can mitigate the negative impact of stress. Among these factors, social support consistently emerges as a key determinant of psychological wellbeing.

## Campus environment, social support, and student wellbeing

The university experience is shaped not only by academic demands but also by the broader campus environment. Zou et al. showed that autonomy, social interactions, and supportive facilities are central to the wellbeing of mid-achieving students, highlighting the value of inclusive policies that meet the diverse needs of students. Social support emerges as a critical protective factor. Research consistently demonstrates that students embedded in strong peer networks, family connections, and institutional support services exhibit greater resilience and more effective coping strategies. These relationships mitigate the effects of psychological distress and contribute to enhanced wellbeing.

While social connections provide a powerful buffer against psychological distress, other contributions focus on targeted interventions designed to actively strengthen resilience and enhance mental health.

## Mindfulness-based and strengths-oriented interventions

Emerging research highlights the value of mindfulness-based and strengths-oriented approaches as integral components of student mental health promotion in higher education. González-Martín et al., through a systematic review and meta-analysis, reaffirmed the effectiveness of these mindfulness programs in enhancing resilience and reducing distress in university students. Chim et al. showed that embedding positive psychology practices into university curricula fosters posttraumatic growth, psychological flexibility, and socio-emotional competencies. Liu N. et al. showed that trait mindfulness predicts prosocial behavior, which is mediated by psychological capital and moderated by moral identity. Merlin and Soubramanian identified intricate links between the intra- and interpersonal dimensions of emotional intelligence, suggesting targeted pathways for skill development. Chang et al. associated structured nature-based experiences with reductions in physiological stress and self-reported anxiety. Hu et al. proposed a positive clinical psychology framework for campus mental health services that integrates preventive, remedial, and strength-based strategies. Qiu et al. revealed that labor education participation enhances subjective wellbeing via self-efficacy and healthy lifestyle adoption. Huang et al. evaluated a Nintendo Switch–based exergame for students with subthreshold depression, aiming to boost mental health and engagement.

## Digital technology and wellbeing

The role of digital technology is complex and multifaceted. While online platforms can increase access to mental health resources and social connections, some patterns of digital media usage are linked to psychological distress. Mei et al. and Ruan et al. reported that self-objectification and body-related discourse on social media increase appearance-related anxiety. Ruini et al. introduced the “DigiWell” protocol, which combines an online positive psychology program with virtual reality in a stepped-care model for varying distress levels. Schaab et al. found that a self-compassion-based mobile app yields mental health benefits and high acceptability among Brazilian students. Liu Y. et al. suggested that digital behavior patterns, such as the timing and duration of online activity, may serve as early indicators for mental health screening. Xu and Li (a) identified that upward social comparison is a predictor of social anxiety, which is mediated by relative deprivation and rumination.

## Cultural and demographic perspectives

A global approach is evident in studies examining wellbeing across diverse socio-cultural and demographic contexts. Xu and Li (b) investigated the impact of perceived discrimination on psychological crises among Chinese college students, identifying life meaning and self-esteem as mediators. Kwok et al. employed a longitudinal, person-centered approach to explore stability and change in meaning-in-life profiles, finding that meaning-oriented profiles are associated with higher wellbeing and lower distress. Lv et al. demonstrated that personal values are related to life satisfaction through the mediating effects of resilience and emotional intelligence. Shifting the focus from psychological resources to labor market perceptions, Zheng and Yan compared the employment psychology of Chinese university students between the initial and normalization stages of the control of the COVID-19 pandemic, showing how shifts in employment attitudes influence job intentions and preferences. Lee et al. validated the Self-Consciousness Type Scale, identifying four distinct profiles with different mental health patterns and offering a novel tool for targeted wellbeing interventions. In these lines, Yang et al. showed that self-control predicts learning engagement, with resilience and positive emotions mediating this relationship. Similarly, Zhang found that academic self-concept and teacher support foster wellbeing, with engagement as a partial mediator and teacher support retaining a direct positive effect.

## Looking ahead: toward holistic mental health in higher education

The studies included in this Research Topic collectively underscore the need for holistic, multi-level approaches to improving psychological wellbeing in university settings. Integrating mental health education into curricula, expanding counseling accessibility, fostering community connections, and promoting balanced digital engagement can create healthier university environments. Ongoing research to optimize these interventions, evaluate their cost-effectiveness, and adapt them to diverse socio-cultural contexts will be pivotal for large-scale implementation.

In conclusion, the contributions to this Research Topic deepen our understanding of the dynamic factors that shape psychological wellbeing in higher education. These findings offer valuable guidance for educators, policymakers, and mental health professionals striving to improve student mental health outcomes amid increasingly complex educational challenges.

